# *KNOX* Genes Were Involved in Regulating Axillary Bud Formation of *Chrysanthemum* × *morifolium*

**DOI:** 10.3390/ijms24087081

**Published:** 2023-04-11

**Authors:** Qingqing Yang, Tianci Cong, Yicen Yao, Tangren Cheng, Cunquan Yuan, Qixiang Zhang

**Affiliations:** 1 Beijing Key Laboratory of Ornamental Plants Germplasm Innovation & Molecular Breeding, Beijing 100083, China; 2 National Engineering Research Center for Floriculture, Beijing 100083, China; 3 Beijing Laboratory of Urban and Rural Ecological Environment, Beijing 100083, China; 4 Engineering Research Center of Landscape Environment of Ministry of Education, Beijing 100083, China; 5 Key Laboratory of Genetics and Breeding in Forest Trees and Ornamental Plants of Ministry of Education, Beijing 100083, China; 6 School of Landscape Architecture, Beijing Forestry University, Beijing 100083, China

**Keywords:** *Chrysanthemum* × *morifolium*, Axillary bud, AM, *KNOX* genes

## Abstract

Branching is an important agronomic and economic trait in cut chrysanthemums. The axillary meristem (AM) formation of the axillary buds of cut chrysanthemums has a decisive role in its branching characteristics. However, little is known about the regulation mechanism of axillary meristem formation in chrysanthemums at the molecular level. Members of the Homeobox gene family especially genes belonging to the class I KNOX branch play a key role in regulating the axillary bud growth and development processes of plants. In this study, three genes belonging to the class I KNOX branch, *CmKNAT1*, *CmKNAT6*, and *CmSTM* were cloned from chrysanthemums, and their functions in regulating axillary bud formation were examined. The subcellular localization test showed that these three *KNOX* genes were expressed in the nucleus, so all of them might function as transcription factors. The results of the expression profile analysis showed that these three *KNOX* genes were highly expressed in the AM formation stage of axillary buds. Overexpression of *KNOX* genes result in a wrinkled leaf phenotype in tobacco and *Arabidopsis*, which may be related to the excessive division of leaf cells, resulting in the proliferation of leaf tissue. Furthermore, overexpression of these three *KNOX* genes enhances the regeneration ability of tobacco leaves, indicating that these three *KNOX* genes may participate in the regulation of cell meristematic ability, thus promoting the formation of buds. In addition, the results of fluorescence quantitative testing showed that these three *KNOX* genes may promote the formation of chrysanthemum axillary buds by promoting the cytokinin pathway while inhibiting the auxin and gibberellin pathways. In conclusion, this study demonstrated that *CmKNAT1*, *CmKNAT6*, and *CmSTM* genes were involved in regulating axillary bud formation of *Chrysanthemum* × *morifolium* and preliminarily revealed the molecular mechanism of their regulation of AM formation. These findings may provide a theoretical basis and candidate gene resources for genetic engineering breeding of new varieties of cut chrysanthemums without lateral branches.

## 1. Introduction

Transcription factors encoded by genes containing homeodomain are widely involved in various growth and development processes of organisms [1]. Homeodomain can play its biological function by combining DNA and it is a three-helical structure composed of 60 amino acids. In spatial structure, the first helix and the second helix of the domain form a ring structure, while the second helix and the third helix form a helix–turn–helix structure [2,3]. Vollbrecht, E et al. found a gene containing homeodomain in plants for the first time, namely the *Knotted-1 (Kn1)* gene of maize. The insertion of transposable elements led to mutations in *Kn1* gene, which seriously affected the development of maize leaves [4]. Genes containing homeodomain have been classified in many ways by predecessors [5,6,7]. One of them is to divide these genes into two categories, namely three amino acid length extension (TALE) and non-TALE [8]. Compared with non-*TALE* genes, the Homeobox domain of the *TALE* genes contain three additional amino acids, which can further form a circular connection in space, which is also the reason for its name [9]. In plants, the *TALE* genes can be further divided into two categories, namely the *BELL (BEL-Like)* genes and the *KNOX (KNOTTED-like Homeobox)* genes [10,11,12].

The transcription factor encoded by the *KNOX* gene usually contains four domains, namely, TALE-type homeodomain Homeobox_KN, KNOX1 domain, KNOX2 domain, and ELK domain. KNOX1 and KNOX2 domains are collectively referred to as the MEINOX domain, which is located at the N terminal of the gene, Homeobox_ KN is located at the C terminal of the gene, while ELK domain is located at the upstream of KN domain [13,14]. At first, people divided the *KNOX* gene into two categories according to its expression profile in plant tissues, including class I KNOX and class II KNOX. Specifically, the expression range of genes belonging to the class II KNOX branch is wide, while the expression of genes belonging to the class I KNOX branch is more tissue-specific [15]. Later, researchers found a type of *KNOX* gene with serious domain deletion in eudicots, that is, only containing KNOX1 or KNOX2 domain, but can regulate the function normally, so people classified it as a new branch, class III KNOX, and this type of *KNOX* gene is unique to eudicots [16]. The class I KNOX branch of *Arabidopsis* contains four genes, namely *STM*, *KNAT1/BP*, *KNAT2*, and the *KNAT6* gene. The expression profile results showed that the expression sites of these genes were mainly distributed in meristem and stem tissues, but not in leaf tissues [17]. *ASYMMETRIC LEAVES1 (AS1)* and *AS2* genes expressed in leaves could inhibit the function of *KNAT1* and *KNAT2* genes to maintain the normal development of leaves, and the ectopic expression of *KNAT1* and *KNAT2* genes in leaves would lead to abnormal leaf development [18]. The *STM* gene, the marker gene of the meristem, is the first *KNOX* gene that can be detected during the development of *Arabidopsis* embryos and plays a key role in regulating the development and maintenance of SAM [19,20]. In addition, similar to *STM* gene, *KNAT1/BP* and *KNAT6* genes are also involved in maintaining the meristematic ability of SAM and regulating organ separation [21,22]. In addition, in the reproductive stage, *KNAT1/BP* gene regulates the expression of *KNAT6* and *KNAT2* genes in the correct position, which is necessary for the normal development of inflorescence [23]. The class II KNOX branch of *Arabidopsis* contains four genes, namely *KNAT3*, *KNAT4*, *KNAT5*, and the *KNAT7* gene, but their expression sites and functions are different from those of the class I KNOX branch. Researchers found that *KNAT7* and its homologous genes could regulate the development of secondary cell wall (SCW) by affecting the synthesis of lignin. At the same time, the *KNAT3* gene can also promote the development of SCW and could play a regulatory role by interacting with the *KNAT7* protein [24,25,26]. In addition, *KNAT3*, *KNAT4*, and *KNAT5* genes and their homologous genes participate in the regulation of root development and the process of symbiotic nodule organ development, and the functions of these genes are redundant [27,28].

The plant type depends on the development of the axillary branches during the post-embryonic development process and the axillary branches develop from the axillary buds, which originate from the axillary meristem (AM) in the center of the leaf axil [29]. It can be seen from the above that the genes of the class I KNOX branch are involved in the regulation of cell meristematic ability [30]. Some studies have found that these genes not only regulate the development of SAM but also participate in the formation of AM in the process of post-embryonic development [31]. Similar to SAM, the *STM* gene is also expressed at a high level in AM [32], and the formation of AM depends on the continuous expression of the *STM* gene [33]. From the axil of P3 to P9 (from the 3rd leaf primordia to the 9th leaf primordia) of *Arabidopsis*, the *STM* gene is only expressed in a few cells, which are named meristematic cell lines. In the axil of P10 and more mature leaf, the number of cells expressing the *STM* gene and the expression intensity of the *STM* gene increased significantly, which was accompanied by the initial formation of AM in leaf axils. In addition, if the meristematic cell line is destroyed by laser cauterization technology, AM cannot be formed normally [33]. The expression pattern of the *STM* gene in leaf axil and laser cauterization test showed that AM was produced by a group of meristem cell lines isolated from SAM, which provided strong evidence for the meristem separation model [34]. The STM protein can directly bind to the promoter of the boundary region–specific expression gene *CUC1* to activate its expression, and can also inhibit the expression of *TCP3* and *TCP4* genes that play a key role in leaf cell differentiation to promote the formation of AM, maintain cell meristematic ability and inhibit cell differentiation [35]. In addition, *CUC* genes can also promote the expression of genes of the class I KNOX branch, including *STM*, *KNAT1*, *KNAT2*, and the *KNAT6* gene, to promote the formation of AM [36]. The rice *ORYZA SATIVA Homeobox1 (OSH1)* gene is a homologous gene of the *STM* gene, and the AM formation of the *osh1* mutant is blocked, resulting in the inability to form tillers normally. Therefore, the *OSH1* gene also plays a key role in the AM formation of axillary buds [37].

The most important feature of single-headed cut chrysanthemums is one flower per stem. However, a large number of lateral branches were produced in the production of single-headed cut chrysanthemums, which needs to be avoided by removing the axillary buds manually, which consumes a lot of labor. Inhibiting the formation of AM formation of axillary buds can solve the above production problems from the source, but the current research on AM formation of axillary buds mainly focuses on model plants, while there are few reports on the formation of AM of cut chrysanthemums. The *DgLSL* gene of cut chrysanthemums has been cloned by predecessors, which is homologous to the *LAS* gene of *Arabidopsis* [38]. The antisense transformation of the *DgLsL* gene into the cut chrysanthemum variety ‘Shuho-no-chikara’ can inhibit the formation of axillary buds to a certain extent [39]. In addition, the chrysanthemum *DgLsL* gene may play a regulatory role by regulating auxin and gibberellin pathways [40]. At the same time, previous studies found that high-temperature treatment prevented the formation of axillary bud in the leaf axil of the chrysanthemum variety ‘Modified Mefo’, and only parenchyma cells with no tissue morphology were produced in the leaf axil [41]. However, the AM formation of the axillary bud of cut chrysanthemum needs further research.

In this study, we cloned three classes of I *KNOX* genes that may regulate AM formation of chrysanthemum axillary buds. Then we carried out gene function verification for these three *KNOX* genes, including gene cloning and bioinformatics analysis, subcellular localization test, analysis of expression profile at different development stages and in different tissue, model plant genetic transformation, and exploring the impact of gene overexpression on hormone pathway. Thus, the molecular regulatory mechanism of the chrysanthemum class I KNOX gene on AM formation of axillary buds was preliminarily analyzed in this study. The results may provide a theoretical basis and candidate gene resources for genetic engineering breeding of new varieties of cut chrysanthemums without lateral branches.

## 2. Results

### 2.1. Cloning and Bioinformatics Analysis of Three KNOX Genes in Chrysanthemum

Through gene cloning, we obtained three chrysanthemum *KNOX* genes (*CmKNOX1*, *CmKNOX2*, and *CmKNOX3*) with a total CDS length of 1086 bp, 903 bp, and 1014 bp, respectively.

**Figure 1:**
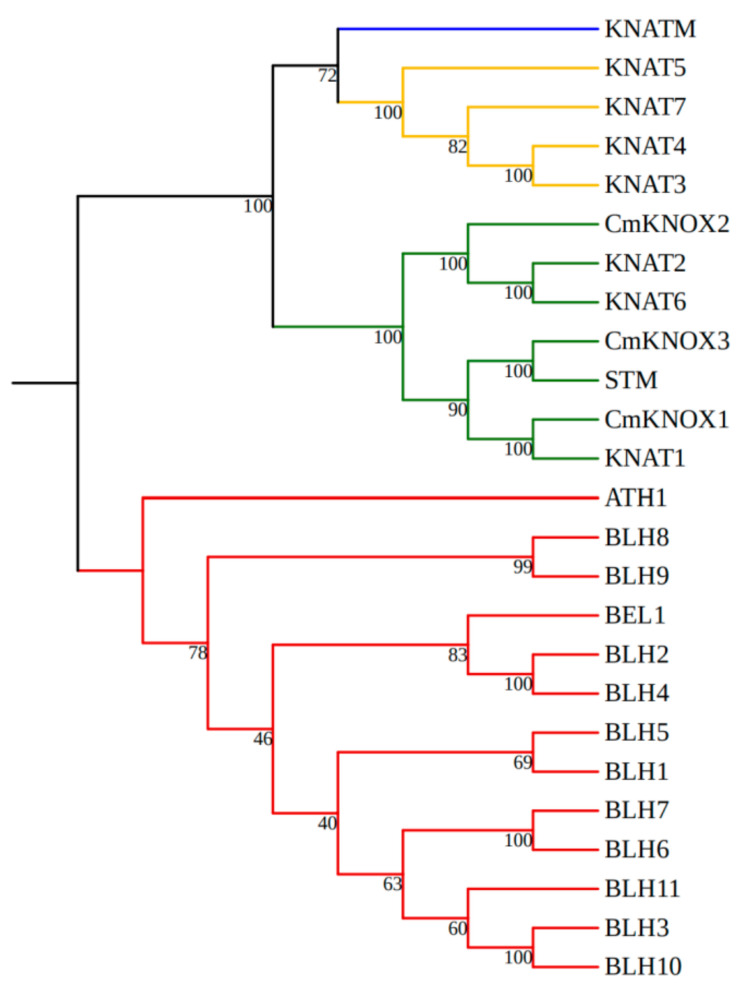
Phylogenetic tree of three chrysanthemum KNOX proteins and *Arabidopsis* TALE gene family members. The red line represents the BELL gene family, which together with the KNOX gene family constitutes the TALE gene family. The green line represents KNOX class I branch, the yellow line represents KNOX class II branch, and the blue line represents the KNATM branch.

The phylogenetic tree was constructed by combining the three chrysanthemums’ KNOX proteins with the members of the *Arabidopsis* KNOX gene family. The results showed that the phylogenetic tree was divided into four evolutionary branches. The red line represents the BELL gene family, which together with the KNOX gene family constitutes the TALE gene family. The green line represents the KNOX class I branch, the yellow line represents the KNOX class II branch, and the blue line represents the KNATM branch. CmKNOX1, CmKNOX2, and CmKNOX3 proteins all belong to the branch of KNOX class I. Among them, CmKNOX1 is clustered with the *Arabidopsis* KNAT1 protein, so it is renamed as CmKNAT1, CmKNOX2 is clustered with *Arabidopsis* KNAT6/AtKNAT2 protein, so it is renamed as CmKNAT6 and, CmKNOX3 is clustered with *Arabidopsis* STM protein, so it is renamed as CmSTM (Figure 1).

**Figure 2:**
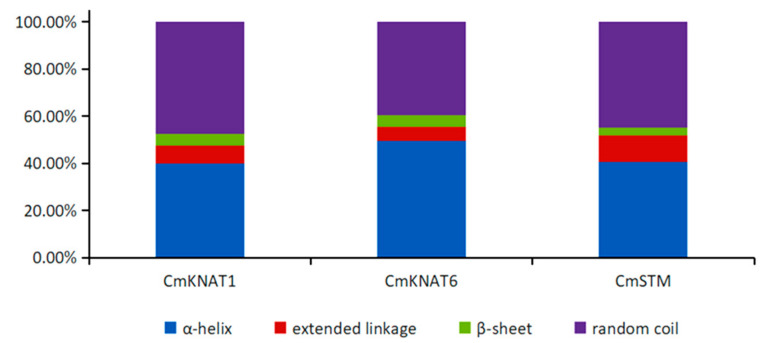
Prediction of secondary structure of KNOX proteins in chrysanthemum.

**Figure 3:**
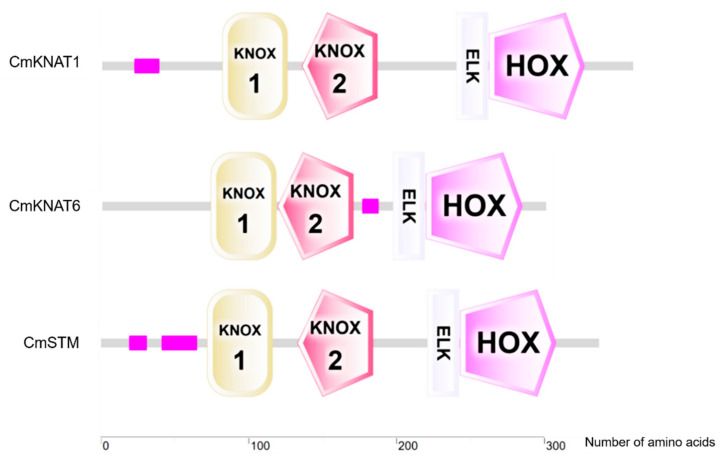
Distribution of conservative domains of KNOX proteins in chrysanthemums.

The sequence analysis showed that the isoelectric points of the three chrysanthemums KNOX proteins were 5.56, 5.03, and 5.72, respectively, which were all less than 7, showing acidity. The total average hydrophilicity coefficient (GRAVY) of the three chrysanthemum KNOX protein sequences is negative, and the instability coefficient is greater than 40, indicating that all proteins are soluble proteins, and the protein structure is unstable. The results of transmembrane domain prediction showed that there was no transmembrane helical region in the amino acid sequence of the three chrysanthemum KNOX proteins, which were all distributed outside the membrane. The subcellular localization prediction results show that the three chrysanthemum KNOX proteins are located in the nucleus, and may play the role of transcriptional regulation as transcription factors (Table 1). The secondary structure prediction results showed that the secondary structure composition of the three chrysanthemum KNOX proteins was similar (Figure 2). The prediction results of phosphorylation sites showed that there were three types of phosphorylation sites in the three chrysanthemum KNOX proteins, namely, serine residues (S), lysine residues (Y), and threonine residues (T), of which serine residues (S) accounted for the highest proportion (Table 2). The results of conservative domain analysis showed that the three chrysanthemum KNOX proteins contained four conservative domains, namely Homeobox_ KN, KNOX1, KNOX2, and ELK domains (Figure 3).

### 2.2. Subcellular Localization of These Three Chrysanthemum KNOX Genes

**Figure 4:**
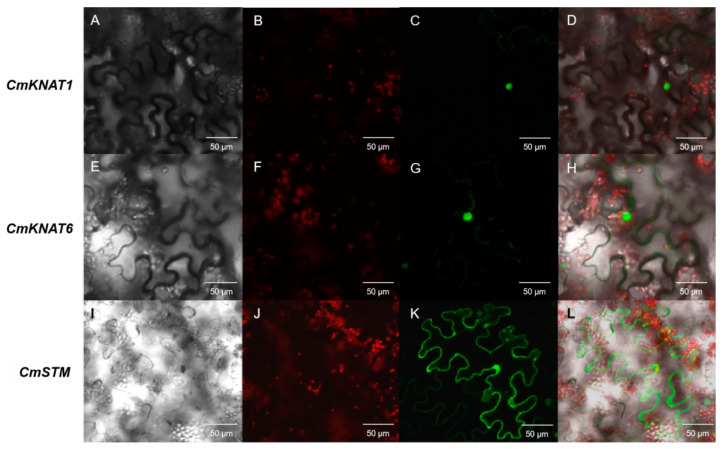
Subcellular localization of these three chrysanthemum *KNOX* genes in *Nicotiana benthamiana* leaves ((**A**,**E**,**I**) show the cells under bright field; (**B**,**F**,**J**) show the spontaneous red light of chloroplasts; (**C**,**G**,**K**) show the green fluorescence of GFP protein; (**D**,**H**,**L**) show the superposition of bright field and fluorescence).

**Figure 5:**
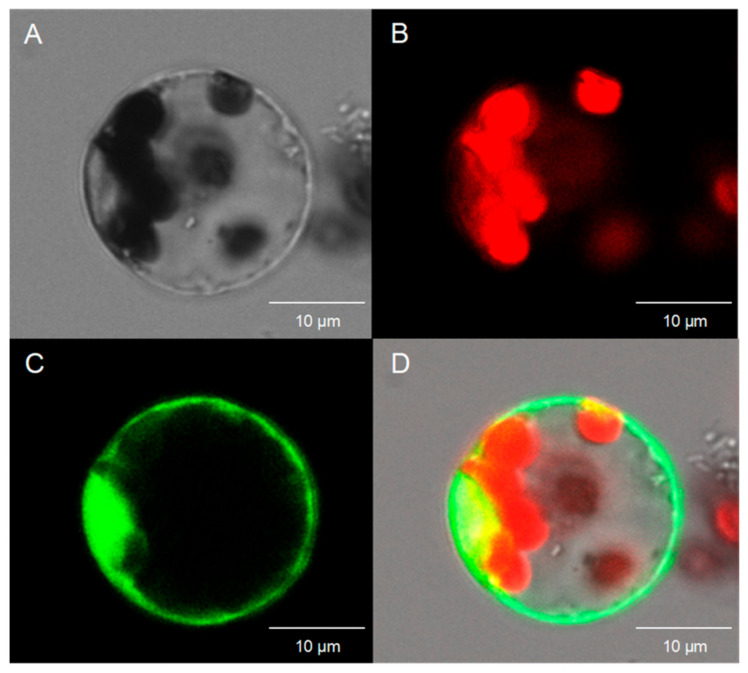
Subcellular localization of chrysanthemum *CmSTM* gene in chrysanthemum protoplast ((**A**) shows the cells under bright field, (**B**) shows the spontaneous red light of chloroplasts, (**C**) shows the green fluorescence of GFP protein, and (**D**) shows the superposition of bright field and fluorescence).

The distribution of chrysanthemum KNOX–GFP fusion protein in tobacco leaf cells was observed by using *Agrobacterium tumefaciens* to transform the tobacco leaves. The results of subcellular localization of *CmKNAT1* and *CmKNAT6* genes showed that the green fluorescence signal of the GFP protein was mainly distributed in the nucleus, and only a small part was distributed in the cytoplasm, indicating that CmKNAT1 protein and CmKNAT6 protein mainly accumulated and functioned in the nucleus. The results of subcellular localization of the *CmSTM* gene showed that the green fluorescence signal of GFP protein was distributed in the nucleus and cytoplasm, indicating that CmSTM protein was significantly accumulated in the nucleus and cytoplasm of tobacco cells (Figure 4). The distribution of chrysanthemum CmSTM–GFP fusion protein in chrysanthemum protoplast cells was observed by a transient transformation of the plasmid. The results showed that the green fluorescence signal of GFP protein was distributed in the nucleus and cytoplasm, indicating that CmSTM protein was significantly accumulated in the nucleus and cytoplasm of chrysanthemum protoplast, which was consistent with the distribution of CmSTM protein in tobacco cells (Figure 5). According to the above subcellular localization results, the three chrysanthemum KNOX proteins can enter the nucleus, indicating that these three proteins may play a regulatory role as transcription factors.

### 2.3. Analysis of the Relative Expression Level of These Three Chrysanthemum KNOX Genes

**Figure 6:**
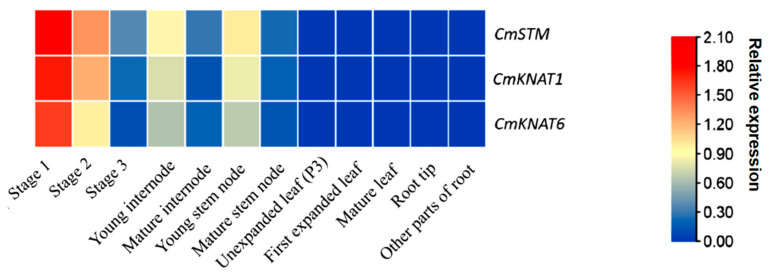
Analysis of the expression profile of these three *KNOX* genes of chrysanthemum at each stage of axillary bud development and each tissue part (Stage 1, Stage 2, and Stage 3 represent the early, middle, and late stages of chrysanthemum axillary bud formation, respectively. The data of each row in this figure has been normalized by the average value of the data of each row, that is, the average value of each row of data is set to 0).

The expression levels of *CmKNAT1*, *CmKNAT6*, and *CmSTM* genes in the different development stages of chrysanthemum axillary bud formation and different tissues were analyzed by fluorescence quantitative PCR. The results showed that the overall situation of the expression profiles of these three chrysanthemum *KNOX* genes was consistent. During the formation of axillary buds, the expression level of these three chrysanthemum *KNOX* genes was continuously down-regulated, that is, the expression level in Stage 1 was high, the expression level in Stage 2 was down-regulated, and the expression level in Stage 3 was further down-regulated. Among them, Stage 1 is the AM formation stage of the chrysanthemum axillary bud and Stage 2 is the leaf primordium formation stage of the chrysanthemum axillary bud. Stage 3 is in the development completion state of the chrysanthemum axillary bud, and at this time, the leaf primordium significantly elongates, tightly wrapping AM. Therefore, these three chrysanthemum *KNOX* genes may participate in the regulation of AM formation of axillary buds (Figure 6). 

In addition, these three chrysanthemum *KNOX* genes showed low expression levels in leaf tissues at different development stages (including P3, the first expanded leaf, and the mature leaf), further indicating that these three chrysanthemums *KNOX* genes may not participate in the regulation of leaf primordium formation in the middle and late stage of axillary bud formation, but mainly participate in the regulation of AM formation in the early stage of axillary bud formation. In addition, compared with the mature internode and mature stem node, these three chrysanthemum *KNOX* genes present a high level of expression in the young internode and young stem node, indicating that these three chrysanthemum *KNOX* genes may also participate in the regulation of young stem tissue development. However, these three chrysanthemum *KNOX* genes present a low level of expression at the root tip and other parts of the root, indicating that these three chrysanthemum *KNOX* genes may not participate in the regulation of root tissue development (Figure 6).

**Figure 7:**
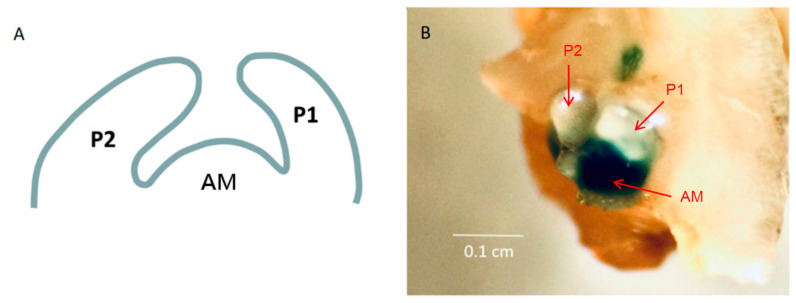
GUS staining results of *CmSTM* gene promoter of chrysanthemum ((**A**) is the schematic diagram of axillary bud structure, and (**B**) is the GUS staining results of an axillary bud of transgenic tobacco; P1 represents the first leaf primordium, P2 represents the second leaf primordium, and AM represents the axillary meristem).

The promoter of the *CmSTM* gene was constructed on PCAMBIASuper1304 to start the expression of the *GUS* gene on the vector, and the constructed vector was transformed into tobacco with *Agrobacterium tumefaciens*, and the axillary buds of transgenic tobacco were stained with GUS staining reagent. The results showed that the meristem region in the center of the axillary bud was stained with indigo blue, while the two leaf primordia on both sides were not stained, indicating that the *CmSTM* gene was almost not expressed in the leaf primordia of the axillary bud, but was highly expressed in the meristem, so it might be involved in the regulation of AM formation (Figure 7).

### 2.4. Phenotypic Observation and Regeneration Test of Model Plants Overexpressing Chrysanthemum KNOX Gene

**Figure 8:**
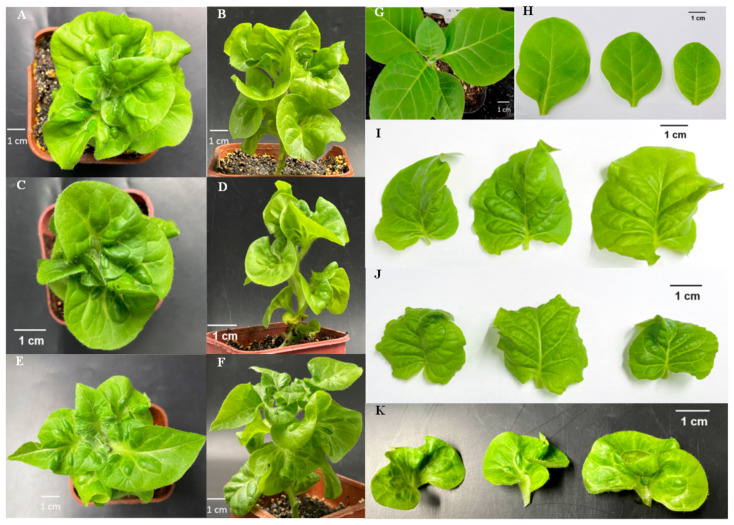
Phenotype observation of tobacco plant overexpressing chrysanthemum *KNOX* gene ((**A**,**B**,**I**) show the top view, front view, and leaves of tobacco plants overexpressing chrysanthemum *CmKNAT6* gene, respectively; (**C**,**D**,**J**) show the top view, front view, and leaves of tobacco plants overexpressing chrysanthemum *CmKNAT1* gene, respectively; (**E**,**F**,**K**) show the top view, front view, and leaves of tobacco plants overexpressing chrysanthemum *CmSTM* gene, respectively; (**G**,**H**) show wild tobacco plants and their leaves).

**Figure 9:**
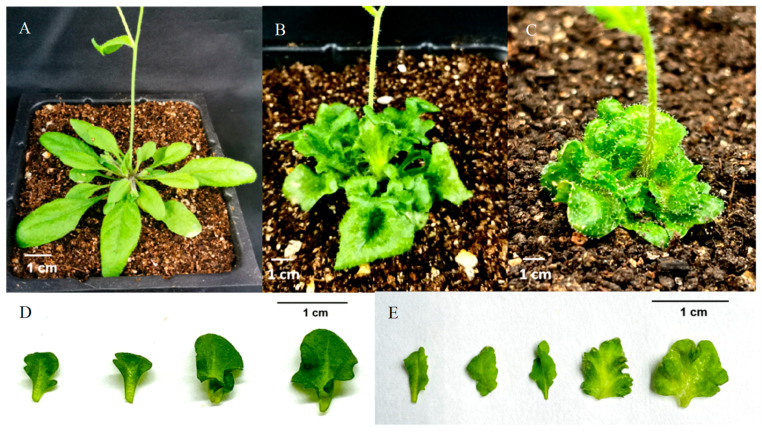
Phenotype observation of *Arabidopsis* plant overexpressing chrysanthemum *KNOX* gene ((**A**) shows wild type *Arabidopsis* plant; (**B**,**D**) show the front view and leaves of *Arabidopsis* plant overexpressing chrysanthemum *CmKNAT1* gene; (**C**,**E**) show the front view and leaves of *Arabidopsis* plant overexpressing chrysanthemum *CmSTM* gene).

The phenotypic observation of the T2 generation of regenerated tobacco plants obtained from tobacco leaf discs infected by *Agrobacterium tumefaciens* showed that compared with wild-type tobacco, transgenic tobacco overexpressing *CmKNAT1*, *CmKNAT6*, and *CmSTM* genes showed abnormal leaf folds (Figure 8). In addition, compared with wild-type *Arabidopsis*, the leaves of transgenic *Arabidopsis* that overexpressed *CmKNAT1* and *CmSTM* genes were wrinkled, which was consistent with the phenotype of the above transgenic tobacco (Figure 9). The wrinkled leaf phenotype of transgenic tobacco and transgenic *Arabidopsis* may be related to the excessive division of leaf cells, resulting in the proliferation of leaf tissue, which is consistent with the fact that *CmKNAT1*, *CmKNAT6* and *CmSTM* genes may be participating in the regulation of cell meristematic ability. 

**Figure 10:**
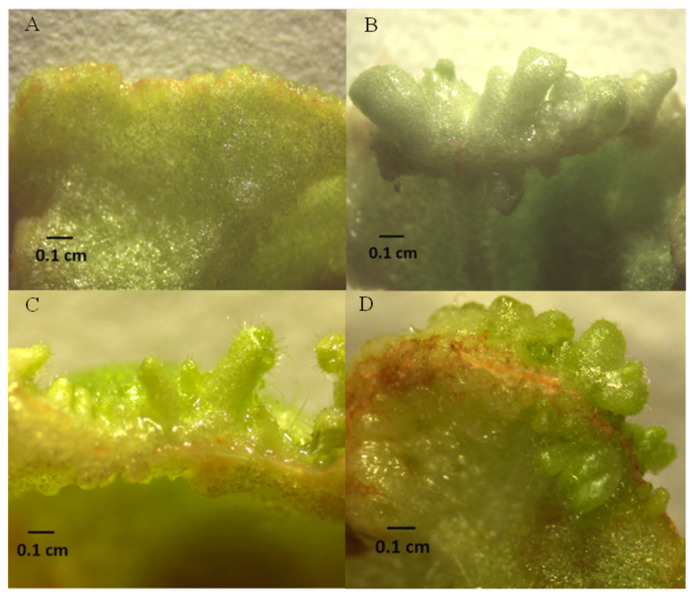
Leaf regeneration test of tobacco plants overexpressing chrysanthemum *KNOX* genes ((**A**) shows the edge of wild-type tobacco leaf tissue after 10 days of tissue culture, (**B**) shows the edge of leaf tissue of tobacco plant overexpressing chrysanthemum *CmKNAT1* gene after 10 days of tissue culture, (**C**) shows the edge of leaf tissue of tobacco plant overexpressing chrysanthemum *CmKNAT6* gene after 10 days of tissue culture and (**D**) shows the edge of leaf tissue of tobacco plant overexpressing chrysanthemum *CmSTM* gene after 10 days of tissue culture).

In order to further verify the function of these three chrysanthemum *KNOX* genes, this study used tobacco leaf tissues overexpressing *CmKNAT1*, *CmKNAT6*, and the *CmSTM* genes as explants for regeneration experiments. The results showed that after 10 days of tissue culture, the edge of the three transgenic tobacco leaves had obvious adventitious buds, while the edge of the wild-type tobacco leaves only expanded, without obvious adventitious buds, This shows that overexpression of *CmKNAT1*, *CmKNAT6*, and *CmSTM* genes makes tobacco leaf tissue have stronger regeneration ability, and further explains that these three chrysanthemum *KNOX* genes may participate in the regulation of cell meristematic ability, thus promoting the formation of bud tissue (Figure 10).

### 2.5. Prediction of the Effect of These Three KNOX Genes on Hormone Pathway

**Figure 11:**
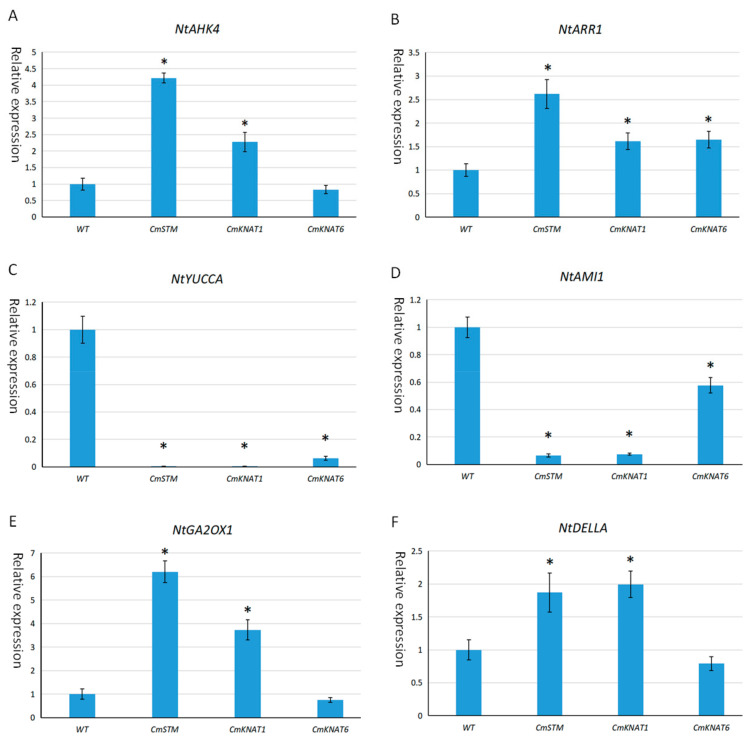
Detection of expression of hormone pathway-related genes in tobacco plants overexpressing chrysanthemum *KNOX* gene (Asterisks indicate a significant difference in gene expression compared to wild-type plants (WT)); (**A**,**B**) show the relative expression of genes related to cytokinin pathway; (**C**,**D**) show the relative expression of genes related to auxin pathway, and (**E**,**F**) show the relative expression of genes related to gibberellin pathway.

In order to further explore the influence of these three chrysanthemum *KNOX* genes on the hormone pathway, this study detected the expression of hormone pathway-related genes in the axillary buds of transgenic tobacco overexpressing *CmKNAT1*, *CmKNAT6*, and *CmSTM* genes, and the results showed that the *NtAHK4* gene encoding cytokinin receptor is up-regulated in the axillary bud tissue of transgenic tobacco overexpressing *CmSTM* and *CmKNAT1* genes, while *NtARR1* gene related to cytokinin signal transduction is up-regulated in the axillary bud tissue of transgenic tobacco overexpressing *CmSTM*, *CmKNAT1*, and *CmKNAT6* genes, which indicates that these three chrysanthemum *KNOX* genes may promote cytokinin pathway. In addition, *NtYUCCA* and *NtAMI1* genes related to auxin synthesis were down-regulated in the axillary bud tissues of transgenic tobacco overexpressing *CmSTM*, *CmKNAT1*, and *CmKNAT6* genes, indicating that these three chrysanthemum *KNOX* genes may have inhibitory effects on the auxin pathway. In addition, *NtGA2OX1* gene related to gibberellin decomposition and *NtDELLA* gene encoding gibberellin signal suppressor are up-regulated in the axillary bud tissue of transgenic tobacco overexpressing *CmSTM* and *CmKNAT1* gene, indicating that *CmSTM* and *CmKNAT1* genes may have an inhibitory effect on the gibberellin pathway (Figure 11). Therefore, the three *KNOX* genes of chrysanthemum may promote the formation of axillary buds by promoting the cytokinin pathway and inhibiting the auxin and gibberellin pathway.

## 3. Discussion

Plant type is one of the important quality traits of ornamental plants, and axillary branch development is the decisive factor of plant type. The axillary branch development includes three stages, namely, the formation of axillary buds, the release of axillary buds (or the dormancy of axillary buds), and the elongation of axillary buds [42]. The research on the axillary branch development of the chrysanthemum mainly focuses on the latter two stages, while the research on the formation of axillary buds is less. The formation of axillary buds takes place in the central position of the axil of the leaf primordium, which is a relatively microscopic development process [29]. Therefore, it is necessary to observe this development process with the help of microanatomy. The formation of axillary buds in *Arabidopsis*, tomato, and rice has been studied. In the middle of the axil of the younger leaves (P15-P21) of *Arabidopsis*, a prominent cell mass, namely AM, was observed. In the axil of P22, AM has differentiated into a leaf primordium, while in the axil of P25, AM has grown three leaf primordia, at which time the axillary bud development is complete [43]. The axillary bud development process of wild-type tomatoes is similar to that of *Arabidopsis*, which shows that the axillary bud development degree is higher in the axil of mature leaves, and lower in the axil of younger leaves [44]. The axillary bud development process of monocotyledonous plant rice is similar to the two dicotyledonous plants mentioned above [45]. In this study, the formation process of axillary buds of cut chrysanthemum was divided into three stages, of which the first stage was AM formation stage, and the axillary bud gradually formed as AM differentiation produced leaf primordia. The overall performance is that the proportion of AM decreases gradually and the proportion of leaf primordium increases gradually with the formation of axillary buds. 

Transcription factors have been found to play a key regulatory role in the formation of axillary buds. For example, the CUC transcription factor can promote the formation of axillary buds, the axillary buds in the axil of the *cuc3* mutant are missing, and the mutant phenotype of the *cuc3* and *cuc2* double mutants is more significant [46]. *CUC1* and *CUC2* genes are located at the upstream of *LAS* gene and can directly bind to the promoter of the *LAS* gene to activate its expression. Then the *LAS* gene regulates the formation of axillary buds by promoting the expression of the *REV* gene [47]. In addition, the *RAX1* gene can promote the formation of AM by regulating the expression of the *CUC2* gene [48], and *RAX1* and *LAS* genes can activate the expression of the *ROX* gene, overexpression of which can cause the formation of adventitious buds in leaf axils [49]. Therefore, the transcription factors regulate each other, form a network, and realize precise regulation on the formation of the axillary bud. Considering the importance of transcription factors in this development process, we cloned some KNOX transcription factors in this study, which may be involved in the regulation of AM formation of axillary bud.

*KNOX* genes belong to the KNOX gene family, and their encoded proteins contain four domains, namely the KNOX1 domain, KNOX2 domain, ELK domain, and Homeobox_ KN domain [13,14]. Homeobox_ KN domain has one more ring structure than the conventional homeodomain, which is a characteristic of the TALE gene family [9]. Therefore, *KNOX* genes belong to the TALE branch of the Homeobox gene family. Homeobox_ KN domain can bind the promoter sequence of the downstream gene, and the lk domain encodes the nuclear localization sequence, which may be involved in the regulation of transcriptional inhibition [50]. In addition, the KNOX gene family can be divided into three branches, namely class I KNOX, class II KNOX, and class III KNOX. Among them, the expression pattern of genes belonging to the class I KNOX branch is tissue-specific and may play a key role in the regulation of cell meristematic ability [15]. In this study, we cloned and bioinformatics analyzed the three *KNOX* genes that may be involved in the regulation of chrysanthemum axillary bud formation. The results showed that these three *KNOX* genes all contain the characteristic domain of the KNOX gene family, all belong to the class I KNOX branch, and all can enter the nucleus, so they may also be involved in the regulation of cell meristematic ability as transcription factors.

The regulation of the gene of the class I KNOX branch on the cell meristematic ability is manifested in participating in the development of meristematic tissue. For example, *Arabidopsis STM*, *KNAT1/BP*, and *KNAT6* gene jointly regulate the development of SAM during embryonic development [21,22,30]. In addition, some studies have found that the meristematic cell line persistently expressing the *STM* gene in leaf axil is the precursor cell of AM formation of axillary buds [33], and the *STM* gene can also coordinate with the *WUS* gene to regulate the correct expression of *CLV3* gene in the meristem, thus re-establishing the negative feedback regulatory loop between *WUS* and *CLV3* gene in AM, which is crucial to maintain the stability of the number of meristem cells [51]. Leaf primordia are produced by differentiation of SAM or AM, and no longer have the ability of meristem without external conditions. Studies have shown that some genes can inhibit the expression of *KNOX* genes in leaf primordia, thus maintaining normal leaf development, such as *BOP1 (BLADE ON PETITLE 1)*, *BOP2*, *AS1*, and *AS2* genes [9,52,53,54,55]. In addition, *BOP1* and *BOP2* genes can activate the expression of *AS1* and *AS2* genes [53], and then AS1/AS2 protein complex can continuously inhibit the function of *KNOX* genes in leaf primordia by recruiting chromatin-remolding protein HIRA [56]. Similar to previous research results, we found that these three chrysanthemum *KNOX* genes were highly expressed in the AM formation stage, but hardly expressed in leaf primordia. In addition, transgenic tobacco and *Arabidopsis* overexpressing the chrysanthemum *KNOX* gene showed abnormal fold in leaves, which is because the ectopic expression of the *KNOX* gene in leaves affected the development of leaves, resulting in tissue proliferation of leaves. In addition, the overexpression of these three *KNOX* genes can increase the ability of tobacco leaf explants to regenerate adventitious buds, which again shows that these three chrysanthemum *KNOX* genes may participate in the regulation of AM formation as well as the *KNOX* genes in other species previously reported, thus promoting the formation of axillary buds.

**Figure 12:**
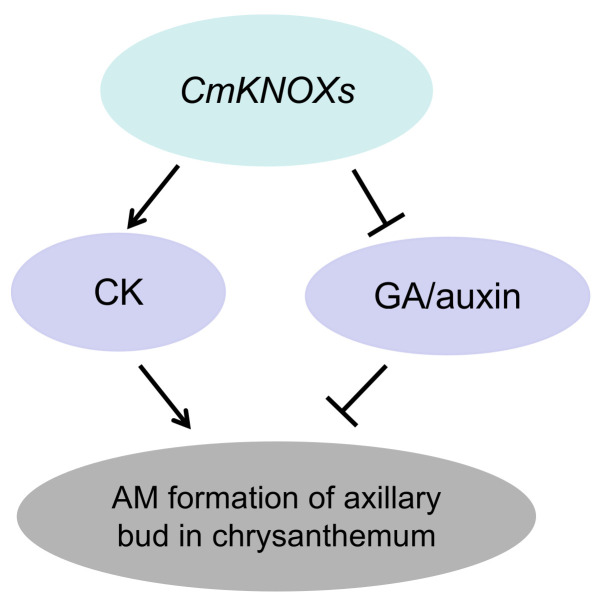
*KNOX* genes regulate axillary bud formation of chrysanthemums by affecting hormone pathways (*CmKNOXs* represent *CmKNAT1*, *CmKNAT6*, and *CmSTM*, CK represents cytokinin, GA represents gibberellin, and AM represents axillary meristem).

*KNOX* genes often exert their regulatory function by influencing the hormone pathway. For example, *KNOX* genes can inhibit the function of GA by down-regulating the expression of genes related to GA synthesis and up-regulating the expression of genes related to GA degradation [57,58,59]. In addition, *KNOX* genes can also maintain the normal development of SAM by activating the gene expression encoding CK biological synthetase [60,61]. The ectopic expression of *KNOX* genes in leaves can affect the auxin signal, thus changing the normal morphology of leaves [62]. In this study, we detected the expression of hormone pathway-related genes in the axillary bud of tobacco overexpressing the chrysanthemum *KNOX* gene and found that GA and auxin signals were down-regulated, while CK signals were up-regulated. Therefore, these three chrysanthemum *KNOX* genes may promote the formation of AM in the axillary bud by promoting the CK signal and inhibiting GA and auxin signal at the same time (Figure 12).

The shortage of single-flower cut chrysanthemum varieties without lateral branches and the traditional production mode of artificial axillary bud removal to achieve one stem and one flower caused the increase of production cost year by year. Therefore, it is a core scientific question to analyze the regulation mechanism of axillary bud formation in breeding new cultivars of cut chrysanthemums without lateral branches. This study revealed the important role of the *KNOX* genes in regulating the axillary bud formation of cut chrysanthemums. Considering the *KNOX* genes mainly function in the early stage (AM formation stage) of axillary bud formation, if the *KNOX* genes can be specifically suppressed or knocked out in AM using gene silencing or gene editing techniques in the future, it might be achieved to inhibit the formation of the axillary bud of cut chrysanthemum from the source, thereby cultivating cut chrysanthemum varieties with fewer or no branches through molecular breeding methods, which can greatly save labor costs for the production of cut chrysanthemum. Therefore, this study may provide a theoretical basis and candidate gene resources for genetic engineering breeding of new varieties of cut chrysanthemums without lateral branches.

## 4. Materials and Methods

### 4.1. Bioinformatics Analysis of Three KNOX Genes in Chrysanthemum

The phylogenetic tree analysis of the chrysanthemum *KNOX* genes: The chrysanthemum *KNOX* genes were analyzed together with the members of the *Arabidopsis* TALE gene family. Sequence information of *Arabidopsis* TALE gene family members from the UniProt (https://www.uniprot.org/blast; accessed on 8 March 2021) website. MEGA7.0 software was used to construct phylogenetic trees of chrysanthemum *KNOX* genes and members of the *Arabidopsis* TALE gene family by neighborhood joining (NJ) and 1000 bootstrap repeats. Among them, the BELL gene family in the *Arabidopsis* TALE gene family serves as an outgroup protein.

Gene sequence and coding protein sequence analysis of chrysanthemum *KNOX* genes: The sequence analysis used an online tool on the Smart website (http://smart.embl-heidelberg.de; accessed on 8 March 2021) was used to compare and analyze the amino acid sequence of chrysanthemum KNOX proteins with the known domain, so as to predict the conservative domain of chrysanthemum KNOX proteins. The isoelectric point (PI), molecular weight (MW), total average hydrophilicity coefficient (GRAVY), and instability coefficient (Instability coefficient) of the proteins encoded by the *KNOX* genes of chrysanthemum were analyzed by using the EXPASSY website (https://web.expasy.org/protparam; accessed on 8 March 2021). The TMHMM 2.0 website (https://services.healthtech.dtu.dk/services/TMHMM-2.0; accessed on 8 March 2021) was used to analyze the transmembrane domain of chrysanthemum KNOX protein. The WOLF PSORT website (https://wolfpsort.hgc.jp; accessed on 8 March 2021) was used to predict the subcellular localization of the chrysanthemum KNOX protein. The SOPMA website (https://npsa-prabi.ibcp.fr/cgi-bin/npsa_automat.pl?page=npsa_sopma.html, accessed on 8 March 2021) was used to predict the secondary structure of chrysanthemum KNOX protein. Prediction of protein phosphorylation sites of chrysanthemum KNOX protein was undertaken using the NetPhos 3.1 website (https://services.healthtech.dtu.dk/services/NetPhos-3.1; accessed on 8 March 2021).

### 4.2. Subcellular Localization of Chrysanthemum KNOX Genes

The chrysanthemum KNOX–GFP fusion expression vector was constructed using PCAMBIASuper1300–GFP vector, and the constructed vector was transformed into the receptive state of *Agrobacterium tumefaciens* GV3101 by the freeze–thaw method. Then *Agrobacterium tumefaciens* was used to transiently transform the chrysanthemum KNOX–GFP fusion expression vector into the lower epidermal cells of tobacco leaves and the fluorescence signals in tobacco leaf cells were observed by fluorescence confocal microscope SP8 after cultured for 24 h and 72 h.

In addition, chrysanthemum protoplast cells were obtained by enzymolysis, and the concentration of protoplast cells was adjusted to 5 × 10^5^ pieces per milliliter. Each 20 mL of enzymolysis solution contains 0.3 g cellulase and 0.08 g macerozyme enzyme. The chrysanthemum KNOX–GFP fusion expression vector was extracted with an endotoxin-free high-purity plasmid mass extraction kit, and the plasmid concentration was adjusted to 1–2 μg/μL. Then the fusion expression plasmid was transiently transformed into chrysanthemum protoplasts by the PEG transformation method. The reaction system was 300 μL protoplast cells, 30 μL plasmid, and PEG4000 solution 330 μL. After the transformed protoplasts were cultured under low light for 12 h, the fluorescence signals in chrysanthemum protoplasts were observed by fluorescence confocal microscope SP8. 

### 4.3. Analysis of the Relative Expression Level of Chrysanthemum KNOX Genes

The relative expression levels of three chrysanthemum *KNOX* genes at different stages of chrysanthemum axillary bud formation and in different tissues were analyzed in this study. The development stages of chrysanthemum axillary bud formation include Stage 1, Stage 2, and Stage 3. Different tissue of chrysanthemum includes apical bud, young stem node, mature stem node, young stem internode, mature stem internode, leaf primordium P3 (unexpanded leaf), the first expanded leaf, mature leaf, root tip, and the rest of the root. Among them, the apical bud refers to SAM and the surrounding two leaf primordium (this is, SAM + P1 + P2). All samples were carefully cut off by the surgical blade and stored in the refrigerator at −80 °C. Then used the EASYSpin Plus trance RNA rapid extraction kit to obtain RNA from each sample and used the PrimeScript RT Reagent kit to synthesize the cDNA of each sample. The fluorescent quantitative primer design was completed by the NCBI primer tool, and the primer sequence information is shown in Table S1. The SYBR Premix Ex Taq II kit was used for fluorescence quantitative reaction. The reaction system (20 μL) includes 10 μL of SYBR Mix, diluted 10 times of cDNA template 1 μL, positive and reverse quantitative primers (10 μmol/L) each 0.4 μL, 8.2 μL H_2_O). The reaction procedure is 95 °C pre-denaturation for 3 min; denaturation at 95 °C for 10 s, and renaturation at 60 °C for 1 min, 40 cycles; fluorescence was collected at 60 °C. The Chrysanthemum *18S* gene was used as an internal reference gene, and three technical repeats were set. The relative expression level of *KNOX* genes in chrysanthemum was calculated by the 2^−ΔΔCt^ formula. The data statistics and picture drawing of fluorescence quantitative results were completed by Microsoft Office Excel. 

### 4.4. Acquisition and Phenotypic Observation of Model Plants Overexpressing Chrysanthemum KNOX Gene

Tobacco transgenic plants overexpressing chrysanthemum *KNOX* gene were obtained by using *Agrobacterium tumefaciens* to infect wild-type tobacco (*Nicotiana tabacum*) leaf explants, and *Arabidopsis* transgenic plants overexpressing chrysanthemum *KNOX* gene were obtained by using *Agrobacterium tumefaciens* to infect inflorescence of wild-type *Arabidopsis* (Col ecotype). Then the phenotypes of T2 generation transgenic tobacco and transgenic *Arabidopsis* plants were observed and recorded. The expression vector used for the stable transformation of model plants is the PCAMBIASuper1300–GFP vector.

The leaves of wild-type tobacco and transgenic tobacco overexpressing chrysanthemum *KNOX* gene were cut into 1 cm × 1 cm squares, then the leaf explants after cutting were sterilized and placed on the regeneration medium for the regeneration test. After 10 days of cultivation under light conditions, the growth of adventitious buds on the edge of the leaves of wild-type and transgenic tobacco overexpressing the chrysanthemum *KNOX* gene was compared, and the photos were taken with a stereomicroscope. The disinfection steps of tobacco leaves are as follows: treatment with 75% ethanol solution for 10 s, cleaning with sterile water once, treatment with 2% hypochlorite solution for 5 min, cleaning with sterile water for 3 times, and drying with filter paper. The formula of 1 L regeneration medium is 7 g agar, 4.43 g MS powder, 25 g sucrose, containing 0.5 mg/L NAA and 1 mg/L 6-BA, pH = 5.8.

### 4.5. Prediction of the Effect of KNOX Genes on Hormone Pathway

The RNA from the axillary buds of wild-type tobacco and transgenic tobacco overexpressing chrysanthemum *KNOX* gene was obtained using the PLANTpure general plant total RNA rapid extraction kit and the sample cDNA was synthesized using the PrimeScript RT Reagent kit. Then, the effect of the overexpression of the *KNOX* gene on the expression level of auxin, cytokinin, and gibberellin pathway-related genes was predicted by a fluorescence quantitative test. For the reaction system and procedure of the fluorescence quantitative test, refer to 2.3. *NtAction* is used as the internal reference gene, and the primers used are shown in Table S2.

## 5. Conclusions

The shortage of single-flower cut chrysanthemum varieties without side branches, and the traditional production mode of artificial axillary bud removal to achieve one stem and one flower has caused an increase in cost year by year, which became a prominent problem restricting the development of the industry. It is a core scientific question to analyze the regulation mechanism of axillary bud formation in breeding new cultivars of cut chrysanthemum without lateral branches. In this study, we cloned three *KNOX* genes (*CmKNAT1*, *CmKNAT6*, and *CmSTM*) from *C. morifolium*. The results of bioinformatics analysis showed that these three *KNOX* genes all had four characteristic domains of the KNOX gene family, all belonged to the class I KNOX branch, and were homologous to the *Arabidopsis KNAT1*, *KNAT6*, and *STM* genes respectively. The proteins encoded by these three *KNOX* genes are expressed in the nucleus, so they may play a regulatory role as transcription factors. In addition, these three chrysanthemum *KNOX* genes are highly expressed in the AM formation stage of axillary buds. The ectopic expression of these three *KNOX* genes in tobacco results in a wrinkled leaf phenotype, which may be related to the excessive division of leaf cells, resulting in the proliferation of leaf tissue, and can enhance the ability of tobacco leaf explants to produce adventitious buds. Therefore, these three chrysanthemum *KNOX* genes may participate in the regulation of meristematic ability to affect AM formation of chrysanthemum axillary buds. In addition, this study provides evidence that these three chrysanthemum *KNOX* genes may regulate axillary bud formation through auxin, CK, and GA pathway. In conclusion, this study demonstrated that *CmKNAT1*, *CmKNAT6*, and *CmSTM* were involved in regulating the axillary bud formation of *C. morifolium*. This study will provide a theoretical basis and genetic resources for breeding new varieties of cut chrysanthemums without lateral branches.

## Figures and Tables

**Table 1:** Sequence analysis of three KNOX proteins in chrysanthemum.

Protein Name	Protein Length	MW	pI	GRAVY	Coefficient of Instability	Subcellular Localization Prediction	Homolog in *Arabidopsis*
CmKNAT1	361	41,484.11	5.56	−0.981	53.59	Nucleus	KNAT1
CmKNAT6	300	34,586.26	5.03	−0.857	44.25	Nucleus	KNAT6
CmSTM	337	37,637.28	5.72	−0.654	48.34	Nucleus	STM

**Table 2:** Prediction of phosphorylation sites of KNOX proteins in chrysanthemum.

Protein Name	Serine Residues (s) Number/Proportion	Lysine Residues (Y) Number/Proportion	Threonine Residues (T) Number/Proportion	Total
CmKNAT1	26 (65%)	9 (23%)	5 (13%)	40
CmKNAT6	23 (56%)	8 (20%)	10 (24%)	41
CmSTM	23 (74%)	4 (13%)	4 (13%)	31

## Data Availability

The original contributions presented in the study are included in the article/Supplementary Material, further inquiries can be directed to the corresponding authors.

## Supplementary Materials

The following supporting information can be downloaded at: https://www.mdpi.com/article/10.3390/ijms24087081/s1.
