# Supplementary material for: KNOX Genes Were Involved in Regulating Axillary Bud Formation of Chrysanthemum × morifolium
Source: Int J Mol Sci. 2023 Apr 11;24(8):7081. doi: 10.3390/ijms24087081 (PMC10138332; doi:10.3390/ijms24087081)
Supplement: Supplementary file 1 [file ijms-24-07081-s001.zip › ijms-2331326-supplementary.docx]

**Supplement**

**Table S1.** Fluorescence quantitative primer sequence for the analysis of the expression profile of *KNOX* genes in chrysanthemum

| **Primer name** | **Primer sequence** |
| --- | --- |
| CmSTM-qPCR-F | ATTGCGCAAGTATAGCGGGT |
| CmSTM-qPCR-R | GTCAAGCAACTGTTGGCGAG |
| CmKNAT1-qPCR-F | AAAGACCCCGAACTTGACCAG |
| CmKNAT1-qPCR-R | GATCCGACGCATGAACTCCA |
| CmKNAT6-qPCR-F | CCACCGGAGATGGTGTGTTT |
| CmKNAT6-qPCR-R | TGCCGATATGTCTCCATGAACTC |
| Cm18S-qPCR-F | AAACGGCTACCACATCCAAG |
| Cm18S-qPCR-R | ACTCGAAAGAGCCCGGTATT |

**Table S2.** Fluorescence quantitative primer sequence for predicting the effect of chrysanthemum *KNOX* genes on hormone pathway

| **Primer name** | **Primer sequence** |
| --- | --- |
| NtAHK4-qPCR-F | AGCGGGAAGGCAGTTCTTAC |
| NtAHK4-qPCR-R | CATGCTCAGTCGGGTTTTGC |
| NtARR1-qPCR-F | CTTCCCTCAACGGGCTTGAT |
| NtARR1-qPCR-R | AGCAGATCTACCTAGGGCCG |
| NtYUCCA-qPCR-F | TCCCTCACATGGTCGTCAGA |
| NtYUCCA-qPCR-R | GGCAACCATTTGAGAAGTGCC |
| NtAMI1-qPCR-F | TGGCTTAACCTTTGCCGTGA |
| NtAMI1-qPCR-R | GTTGCAGTAGATGTTGCCGC |
| NtGA2OX1-qPCR-F | AGGTGATGACTAACGGGAGGT |
| NtGA2OX1-qPCR-R | AATGGCGGCCCTCCAAAATA |
| NtDALLE-qPCR-F | GTCGTCGTCCTCTTCAAGCA |
| NtDALLE-qPCR-R | CTTGCGAGTCAACAAGCACC |
| NtActin-qPCR-F | TGTGTTGGACTCTGGTGATG |
| NtActin-qPCR-R | CGCTCGGTAAGGATCTTCATC |
